# The Oesophageal Squamous Cell Carcinoma Cell Line COLO-680N Fails to Support Sustained *Cryptosporidium parvum* Proliferation

**DOI:** 10.3390/pathogens11010049

**Published:** 2021-12-31

**Authors:** Juan Vélez, Liliana M. R. Silva, Faustin Kamena, Arwid Daugschies, Sybille Mazurek, Anja Taubert, Carlos Hermosilla

**Affiliations:** 1 Institute of Parasitology, Biomedical Research Center Seltersberg, Justus Liebig University Giessen, Schubertstr. 81, 35392 Giessen, Germany; Liliana.Silva@vetmed.uni-giessen.de (L.M.R.S.); anja.taubert@vetmed.uni-giessen.de (A.T.); carlos.r.hermosilla@vetmed.uni-giessen.de (C.H.); 2 Institute of Veterinary Physiology and Biochemistry, Justus Liebig University Giessen, Frankfurter Street 100, 35392 Giessen, Germany; sybille.Mazurek@vetmed.uni-giessen.de; 3 Institute for Parasitology, University of Leipzig, An den Tierkliniken 35, 04103 Leipzig, Germany; faustin.kamena@uni-leipzig.de (F.K.); daugschies@vetmed.uni-leipzig.de (A.D.); 4 Laboratory for Molecular Parasitology, Department of Microbiology and Parasitology, University of Buea, Buea P.O. Box 63, Cameroon

**Keywords:** *Cryptosporidium parvum*, cell culture, COLO-680N, immunofluorescence, qPCR

## Abstract

*Cryptosporidium parvum* is an important diarrhoea-associated protozoan, which is difficult to propagate in vitro. In 2017, a report described a continuous culture of *C. parvum* Moredun strain, in the oesophageal squamous cell carcinoma cell line COLO-680N, as an easy-to-use system for *C. parvum* propagation and continuous production of oocysts. Here, we report that—using the Köllitsch strain of *C. parvum*—even though COLO-680N cells, indeed, allowed parasite invasion and early asexual parasite replication, *C. parvum* proliferation decreased after the second day post infection. Considering recurring studies, reporting on successful production of newly generated *Cryptosporidium* oocysts in the past, and the subsequent replication failure by other research groups, the current data stand as a reminder of the importance of reproducibility of in vitro systems in cryptosporidiosis research. This is of special importance since it will only be possible to develop promising strategies to fight cryptosporidiosis and its ominous consequences for both human and animal health by a continuous and reliable methodological progress.

## 1. Introduction

*Cryptosporidium parvum and C. hominis* are recognized as protozoan parasite species causing diarrhoea in humans worldwide for a long time, but only recently, the true relevance as the second most important diarrheal pathogen in young children was revealed [1]. Moreover, for immunosuppressed humans, such as HIV patients, and malnourished infants it can become a life-threatening disease [2,3]. Despite its significance, only one FDA-approved drug, namely nitazoxanide, is available to date, but it lacks efficacy in immunocompromised *C. parvum*-infected patients [4]. It is indisputable that more effective medication is urgently required [5]. Therefore, research on *C. parvum*, especially on its biology, invasion, and intracellular survival related mechanisms is crucially needed.

A major problem in *Cryptosporidium* research is the lack of appropriate in vitro systems that enable continuous *C. parvum* culture including oocyst generation. Even though some important tools for basic research on *C. parvum* have recently been achieved by various laboratories around the world, the lack of an easy and reproducible, continuous in vitro culture of this parasite remains the major issue and mainly relies on a failure of sexual reproduction (gamogony) resulting in poor or absent oocyst production. Likewise, in HCT-8 cells, a block of *C. parvum* development at the fertilization step was demonstrated [6]. In principle, developmental achievements in *C. parvum* in vitro propagation were reported for several cell lines, such as bovine fallopian tube epithelial cells BFTE, human Caco-2 cells, MBDK, HCT-8, and HFL [7,8,9]. Although those cell lines seem to support the full life cycle of *Cryptosporidium*, the yield of freshly produced infectious oocysts was minimal and insufficient for reinfection experiments. In most studies, the growth of *C. parvum* was recorded to peak at 48–72 h *post infectionem* (hpi) and then to gradually decline. In newly developed multifactorial culture systems, such as organoid- or stem cell-based air- liquid-cell cultures [10,11,12], *C. parvum* seems to replicate considerably over time. However, the complexity of these cultures hampers easy utilization and reproducibility. A promising progress in easy-to-propagate cultures was recently reported by Miller et al. (2018) using the permanent cell line COLO-680N [13]. This cell line was originally isolated from an oesophageal squamous-cell carcinoma in 1985. Miller et al. (2018) showed that *C. parvum* (Moredun strain) can be propagated in COLO-680N cells, by weekly exchange of cell medium, for as long as eight weeks without the necessity of sub-culturing. In addition, it was reported that newly generated, infective oocysts are successfully formed in COLO-680N cells at a considerable amount and can easily be collected from cell culture supernatants to sustain propagation of this parasite in standard tissue cultures at a laboratory scale [13]. Given that this cell culture system seemed extremely promising for any kind of *Cryptosporidium*-related research, we intended to reproduce the data of Miller et al. (2018) in the current work using the Köllitsch strain of *C. parvum*. Thus, we report on successful sporozoite invasion, intracellular trophozoite and meront development, but we also show a failure in gamogony, obviously leading to a lack of considerable oocyst production in COLO-680N-infected cells.

## 2. Results

**Figure 1:**
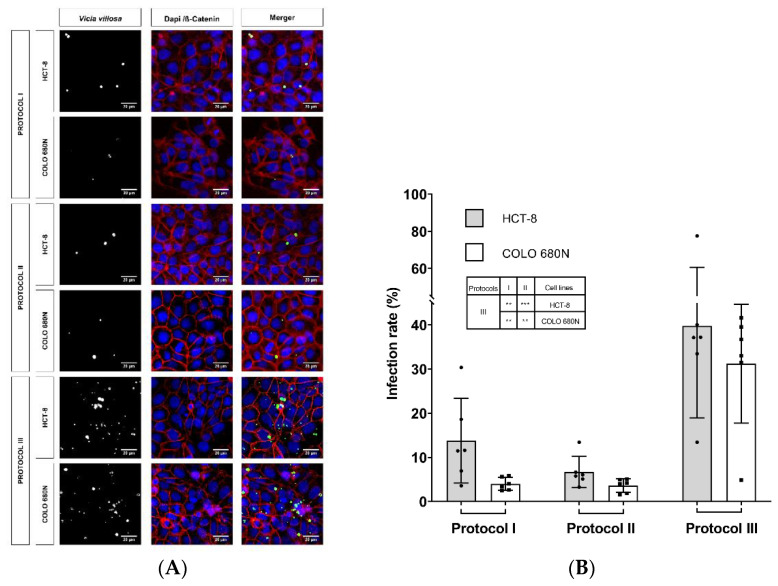
Exemplary illustration of *Cryptosporidium parvum* in vitro cell cultures at 24 hpi (**A**) *Vicia villosa* (VV) lectin-based detection of *C. parvum* (green), illustration of cell membranes via anti-β-catenin-mediated staining (red) and of cell nuclei via DAPI staining (blue). Scale bar 20 µm. (**B**) Infection rates of *C. parvum*-infected HCT-8 and COLO-680N cells after application of three different infection protocols. Infection rates are expressed as mean ± SD of six replicates per condition. For statistical analysis, a one-way analysis of variance (ANOVA) with Tukey’s test was performed using GraphPad^®^ Prism 8 software (San Diego, CA, USA), with a significance level of 5%. The significance levels are as follows: *** = *p* ≤ 0.001, ** = *p* ≤ 0.01 and presented within the table in (**B**).

**Figure 2:**
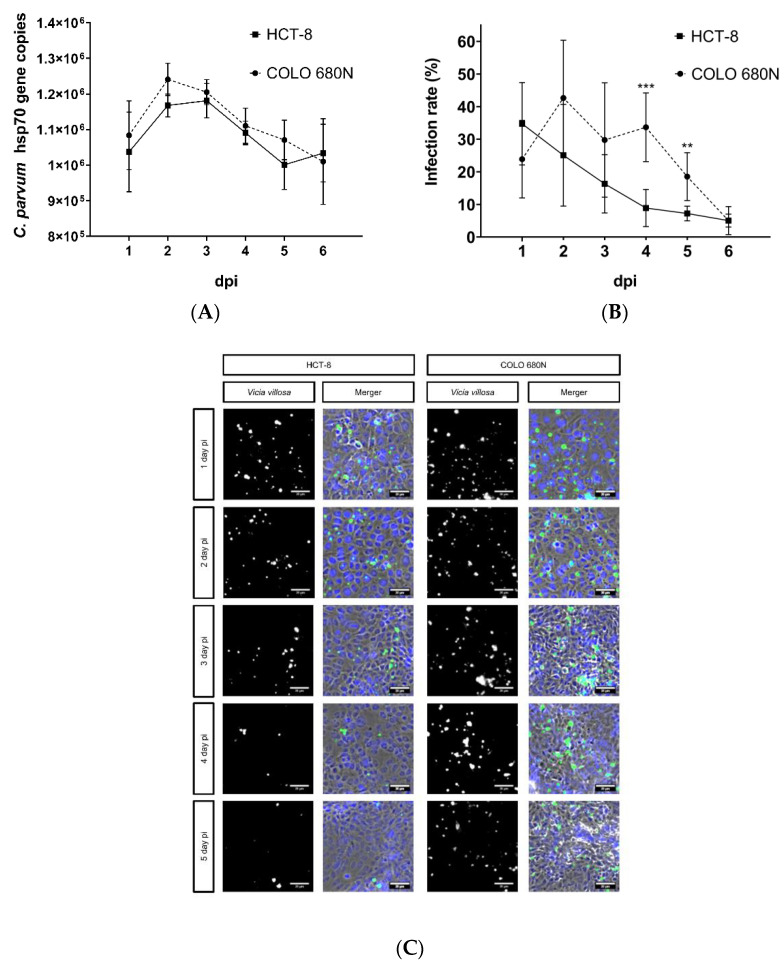
Replication of *C. parvum* in HCT-8- and COLO-680N cells using infection protocol III. Parasite intracellular replication was quantified by both qPCR- (**A**) and *Vicia villosa* (VV) lectin (VVL)-based immunofluorescence analyses (**B**). (**C**) Exemplary illustration of both in vitro cell culture systems: fluorescence-based detection of *C. parvum* via VVL (green) and cell nuclei via DAPI (blue). To assess parasite development, a two-tailed *t*-test was performed, comparing the infection rate per day, measured in HCT-8- and COLO-680N cells. The significance values were as follows: *** = *p* ≤ 0.001, ** = *p* ≤ 0.01. Scale bar 20 µm.

For comparative reasons, *C. parvum* infections were studied in parallel in COLO-680N and HCT-8 cells (HCT-8 cells represent the cell line mostly used in *Cryptosporidium*-related in vitro cultures) and subjected to three different infection protocols, according to Miller et al. (2018) and other publications [14,15]. Here, we used the Köllitsch strain, which was characterized as subgenotype 60-kDa glycoprotein (gp60) IIaA15G2RI of *C. parvum*, the most common zoonotic subgenotype in Germany [16,17]. Overall, similar infection rates were observed in both cell lines (HCT-8 and COLO-680N), applying three different infection protocols (Table 1) and estimating infection rates at 24 hpi by *Vicia villosa* (VVL)-based immunofluorescence (protocol I: HCT-8: 13.76 ± 9.58, COLO-680N: 3.99 ± 1.47; protocol II: HCT-8: 6.71 ± 3.49, COLO-680N: 3.60 ± 1.53; protocol III: HCT-8: 39.81 ± 20.86, COLO-680N: 31.21 ± 13.46) (Table 1; Figure 1). As a general but insignificant trend, slightly higher infection rates were observed in HCT-8 when compared to COLO-680N at 24 hpi (Figure 1; Figure 2). When considering the three different infection protocols, the lowest infection rates were achieved by the protocol of Miller et al. (2018) and Shahiduzzaman et al. (2009) (no statistical difference between these two protocols), whilst a significant higher infection rate was obtained when applying the protocol of Varughese et al. (2014) in both HCT-8 and COLO-680N cells (protocol III against protocol I: at both HCT-8 and COLO-680N: *p* ≤ 0.01; protocol III against protocol II: HCT-8: *p* ≤ 0.001 and COLO-680N: *p* ≤ 0.01). Worth noting, the use of excystation medium with NaTC (protocol II) resulted in enhanced host cell toxicity and/or a block in host cell replication (data not shown).

Given that the protocol of Miller et al. (2018) resulted in poor infection rates, we decided to proceed experimentation with the protocol that worked the best, namely protocol III (Figure 1). Ongoing *C. parvum* replication was evaluated in parallel in both HCT-8- and COLO-680N cells, applying VVL-based fluorescence and qPCR for parasite quantification until 6 dpi. Overall, both cell lines showed an initial increase in parasite replication (as being reflected by enhanced numbers of host cells being infected), comprising the asexual replication (merogony) and formation of mature meronts. However, in contrast to reports for COLO-680N (Miller et al., 2018), no sustained parasite replication or development was observed in this host cell system. As with the well-documented parasite kinetics in HCT-8 cells [6,18,19], the number of infected cells dropped from day 2 onwards, in both cell lines, thereby reflecting incomplete sexual replication and unaccomplished karyogamy as it has been previously described [6,18]. Interestingly, a minimal but significantly higher infection rate was observed in COLO-680N cells at 4 and 5 dpi, in comparison to HCT-8, when quantifying *C. parvum* by VVL-based fluorescence. However, when testing by qPCR, no significant differences were detected at any time point (Figure 2).

## 3. Discussion

The uniqueness of *C. parvum* and the high risk of infection for long-lasting infectivity, in immunocompromised individuals and neonates, relies on both its capacity to continuously replicate via meronts I and its developmental characteristics to perform sporulation already in the gut of infected hosts, thereby leading to the formation of potentially autoinfective thin-walled oocysts [20,21,22]. However, the failure to mimic the latter characteristics in vitro and maintain parasite replication in cell culture, as it occurs in patients with impaired immune system, has been a significant handicap in cryptosporidiosis research since the first report on a *Cryptosporidium* ssp.-related in vitro culture system [23]. In this sense, during the last decades, *C. parvum* in vitro propagation was mainly based on 2D-cell culture systems being restricted to initial asexual replication and meront I/II maturation [6,11,18,24]. Most of these studies have been performed using the permanent human intestinal adenocarcinoma HCT-8 cell line and led to fundamental knowledge on *C. parvum* biology and parasite-host cell interactions, in addition to the identification of potential anti-cryptosporidial drugs [15,18,20,24,25,26]. In 2015, the complete life cycle of *C. parvum* including sustained oocyst production was reported to be accomplished using the hollow fibre technology, simulating the 3D complexity of the small intestine [11]. Nevertheless, even though this system seemed to solve sexual replication-related issues, it also delivered other limitations, due to a highly expensive and complex technology, hampering high-throughput experimentation (necessary for drug development) and impeding the direct observation of parasite-host cell interactions seen *in vivo*. More recently, a human oesophageal squamous-cell carcinoma cell line (COLO-680N) was reported to support the total *C. parvum* life cycle in 2D-cell culture, thereby also allowing for sustained sexual replication and production of infective oocysts [13]. Given that this seemed to be a very promising and easily reproducible approach, we comparatively analysed HCT-8 cells, representing the mostly used cell line in *Cryptosporidium* research and the newly reported COLO-680N cell line. We found an almost similar parasite replication pattern, by means of VVL-immunofluorescence and qPCR, but it was a failure of sustained replication or oocyst production in both cell lines. 

Using three different infection protocols, we were unable to reproduce sustained *C. parvum* intracellular replication, as previously reported by Miller et al. [13], even when applying diagnostic tools of differing sensitivities (lectin-based immunofluorescence and qPCR). Besides the Miller protocol, we also included and comparatively evaluated two other published protocols for *C. parvum* infection [14,15], using HCT-8 and COLO-680N cells. Obviously, the protocol that induced the highest infection rates (protocol III) was the least toxic, in terms of exposing oocysts to rather aggressive chemicals, such as trypsin, sodium hypochlorite, or sodium taurocholate, with excystation duration being particularly short (20 min against 1 and 3 h for protocols I and II, respectively). Likewise, protocol III seemed to mimic the in vivo situation best since it simulated the natural oral infection pathway, which includes oocyst exposure to both an acidified milieu (in vivo: in stomach) and to basic conditions in its natural niche (in vivo: in small intestine), also considering body temperature and gastrointestinal motility [14,22]. However, applying protocol III and thoroughly exploring kinetics, we could not find any sustained replication of *C. parvum* from 72 hpi onwards, neither in HCT-8 nor in COLO-680N cells. Overall, *C. parvum* development hardly differed in these two cell lines and observed parasite replication in both HCT-8 and COLO-680N cells, which are in agreement with previous studies performed in permanent cell lines [6,18,19,20,24]. Moreover, significant differences in infection rates of COLO-680N and HCT-8 cell lines, by means of VVL-immunofluorescence, were possibly caused by non-specific-labelling of polypeptides left behind by parasitic stages in their intracellular but extracytoplasmatic locations. Those differences are probably coupled to distinct clearance capabilities of the studied host cell lines. The above speculation arises from non-significant differences in the number of *C. parvum*-specific gene copies, constituting a disparity in parasite replications observed by VVL-immunofluorescence- and qPCR-based methodologies. However, immunofluorescence and qPCR-derived results are not mutually exclusive, since both revealed the same general pattern of reduction in parasite numbers over time. Our overall findings contrast with previously reported capacity of COLO-680N cells to support sustained *C. parvum* replication [13]. It remains unclear whether these divergent results are due to different parasite strains since Miller et al. (2018) used the Moredun strain [13], whilst we worked with the *C. parvum* Köllitsch strain (IIaA15G2RI), a zoonotic-relevant subgenotype most commonly found in industrialized countries, as reported elsewhere [16,17,27,28]. Although, the last decade has seen the successful sequencing of different *C. parvum* isolates (e.g., IOWA, TU114) the sequences of the Moredun and Köllitsch strains are still not available. Therefore, it hinders a direct comparison on the genomic basis between these two *C. parvum* strains. It is worth mentioning that *C. parvum* development occurs in an extremely complex intestinal niche, consisting of rapidly replicating enterocytes, which evolve from intestinal crypt-derived stem cells that generate highly specialized cell types, such as epithelial, paneth, enteroendocrine, tuft, and goblet cells [29] and which are in constant contact with an equally highly complex and variable microbiome [30]. Indeed, *C. parvum* replication may not only be supported by epithelial host cells but also influenced by non-infected bystander cells, bacterial, parasitic, and/or fungal molecules, as it was previously suggested [31,32]. Obviously, these intrinsic factors of the gut are lacking in commonly applied in vitro cultures. Moreover, genetic and biochemical findings demonstrated that *C. parvum* exclusively relies on glycolysis as an energy-producing pathway [18,33,34]. The resulting metabolic requirements seem to perfectly match to the parasite ileum niche, which represents the main area of carbohydrate absorption in vivo [35] and gut-typical low oxygen concentrations (physioxia) [36], favouring glycolytic responses, which may result in high lactate production found in *C. parvum*-driven metabolic conversion rates [37] and *C. parvum*-infected calves [38,39]. Consequently, parasite-own metabolic capability, its highly specialized natural niche, the potential influence of current microbiomes/macrobiomes (parasites), and physiological intestinal oxygen conditions should be considered in the future to create more realistic culture systems for total *C. parvum* development. As an example, a new ex vivo *C. parvum* system, based on bovine small intestinal (BSI) explants under physioxic (5% O_2_) conditions, might offer a more realistic platform to address pending questions on *C. parvum*-host cell-intestinal microbiome interactions [40]. 

## 4. Materials and Methods

### 4.1. Host Cell Lines and Cell Culture Conditions

COLO-680N and HCT-8 cells (ATCC CCL-244™) were maintained at 37 °C and 5% CO_2_ using RPMI 1640 medium (R0883, Sigma-Aldrich, St. Louis, MI, USA) supplement with 0.3 g/L _L_-glutamine (Sigma-Aldrich), 10% foetal bovine serum (FBS, S0115, Biochrom GmbH, Berlin, Germany), 100 UI penicillin, and 0.1 mg streptomycin/mL (Sigma-Aldrich). The cell culture medium was changed every other day. Cells from the same passage were used for all infection assays. 

### 4.2. Parasites 

*C. parvum* oocysts were obtained from experimentally infected calves (Institute of Parasitology, Leipzig University, Germany) [15]. We used the *C. parvum* Köllitsch strain, which belongs to the most common zoonotic subgenotype in Germany. Oocyst stocks were preserved in sterile phosphate buffered saline (PBS 1X , pH 7.4; Sigma-Aldrich) with 100 UI penicillin, 0.1 mg streptomycin/mL (P4333; Sigma-Aldrich) at 4 °C, for a maximum of three months to guarantee infectivity of parasites. The oocyst conservation medium was renewed monthly, as reported elsewhere [37]. 

### 4.3. Host Cell Infection

Cells were seeded at a density of 1 × 10^5^ cells/well on 10 mm round coverslips, previously treated with 40 µg/mL fibronectin bovine plasma (F1141; Sigma-Aldrich), and deposited in 24-well microtiter plates (Eppendorf, Hamburg, Germany). When cells reached 60–70% confluence, images were taken at 40× magnification, using a phase-contrast microscope (Olympus, IX81 S1F-3) equipped with a digital camera (Olympus XM10, Hamburg, Germany). The total cell number was determined by means of ImageJ^®^ (National Institutes of Health, Bethesda, MD, USA, public domain image analysis software) to calculate the infection dose, applying an MOI (multiplicity of infection) of 0.5 (1 oocyst per 2 host cells). Here, three different infection protocols were used (please refer to Table 1). Initial parasite replication was evaluated until 24 hpi by *Vicia villosa* lectin (VVL)-based staining (VVL; Vector Laboratories, Burlingame, CA, USA) [20,41]. For kinetic analysis covering up to 6 dpi, protocol III was used for host cell infections. In the latter case, intracellular parasite replication was evaluated by both VVL-based fluorescence and qPCR assays.

### 4.4. Vicia villosa Lectin-Based Detection of C. parvum

*C. parvum*-infected cell layers were washed once with sterile 1X  PBS to eliminate remnant oocysts and non-invaded sporozoite stages. Cells were fixed with 4% paraformaldehyde for 15 min and washed with sterile 1X  PBS. Cells were then permeabilized in 0.3% Triton X-100 (T-8787, Sigma-Aldrich) and 3% BSA (1 h treatment; Sigma-Aldrich). For detection of intracellular parasites and posterior calculation of infection rates, staining with the fluorescein labelled *Vicia villosa* lectin (VVL, FL-1231-2, 1:2000, VECTOR laboratories, 45 min, room temperature (RT), dark chamber) was used. Thereafter, cell layers were washed thrice with sterile 1X  PBS. Host cell membranes were additionally labelled by antibodies against ß-catenin (ab32572, Abcam, Cambridge, UK, 1:100, overnight) and secondary antibodies (AlexaFluor-conjugated, Abcam, 594 R37117, 1:500, 45 min, in the dark). The samples were mounted in Fluoromont-G^TM^ with DAPI (Invitrogen, Carlsbad, CA, USA) and allowed to dry for 24 h at RT and darkness. Fluorescence was analysed at 40× magnification by an epifluorescence microscope (Olympus, IX81 S1F-3) equipped with a digital camera (Olympus XM10, Hamburg, Germany). VVL- and DNA-related signals were merged for quantification of infection rates using ImageJ^®^ (National Institutes of Health).

### 4.5. Real-Time qPCR for C. parvum Quantification 

Supernatants of *C. parvum*-infected cell cultures were collected and centrifuged (4000× *g*, 5 min). The supernatants were discarded. Additionally, respective cell layers were washed (1X  PBS), detached by trypsin (2.5%, 5 min) treatments, and pelleted together with the pellet of the respective cell culture supernatant (4000× *g*, 5 min). The resulting cell pellet was resuspended in 300 µL sterile 1X  PBS and frozen at −20 °C for later DNA extraction.

For DNA isolation, each sample was transferred to bead-beating tubes with 2.8 mm ceramic beads (Thermo Fisher Scientific, Waltham, MA, USA). Physical disruption was achieved using a bead-beating machine (Bead ruptor 24^®^, OMNI International, Kennesaw, GA, USA), followed by the silica-membrane-based DNA purification protocol of DNEasy Blood & Tissue Kit^®^ (Qiagen, Hilden, Germany). Briefly, samples were beaten at 1 m/s for 9 cycles of 20 s, with pauses of 10 s between cycles, and then centrifuged at 6000× *g* for 15 s. Thereafter, the supernatant was transferred into a 2 mL microtube, adding 200 µL ATL buffer (Qiagen) and 20 µL proteinase-K (20 mg/mL; Qiagen). The microtubes were vortexed for 15 s and incubated at 56 °C under constant agitation in a thermomixer (Eppendorf AG). The DNA purification protocol for Blood and Tissue (Qiagen) was performed according to manufacturer instructions.

For qPCR, partial gen of the heat shock protein 70 (HSP 70) of *C. parvum* was amplified using the following primers: forward primer: CP_hsp70_fwd 5′-aactttagctccagttgagaaagtactc-3′; reverse primer: CP_hsp70_rvs 5′-catggctctttaccgttaaagaattcc-3′ and TaqMan probe: HSP_70_SNA 5′-aatacgtgtagaaccaccaaccaatacaacatc-3′, as previously reported [15,40]. 

The final PCR reaction volume consisted of 20 μL, containing 10 μL of 1X PerfeCTa qPCR mix (VWR, Darmstadt, Germany), 0.8 μL of forward- and reverse-primer (10 pmol/μL), and 0.4 μL of 200 nM of HSP_70_SNA (Biomers, Ulm, Germany) labelled at the 5′-end with the 6-carboxyfluorescein reporter dye (FAM) and at the 3′-end with the 6-carboxytetramethylrhodamine quencher dye (TAMRA). Reactions were performed on a Rotor-Gene Q^®^ cycler (Qiagen, Hilden, Germany) using the following cycling conditions: 95 °C for 15 min (initial denaturation), followed by 40 cycles of 95 °C for 20 s (denaturation), 54 °C for 30 s (annealing), and 72 °C for 2 min 30 s [40].

For quantification, plasmid DNA of the cloned amplification target (fragment of HSP 70 gene) used for standard curve generation. HCT-8-DNA probes were incorporated to each assay as negative control.

### 4.6. Statistics

Infection rate results are presented as mean ± SD of six replicates for each condition. For evaluation of infection rates obtained through the three applied infection protocols, a one-way analysis of variance (ANOVA) with Tukey’s test was performed, using GraphPad Prism 8^®^ software with a significance level of 5%. For evaluation of parasitic development, by means of protocol III (Figure 2), two-tailed t-test was used, comparing the infection rate observed in HCT-8 and COLO-680N cell lines each day. Significance values are presented as follows: *** = *p* ≤ 0.001, ** = *p* ≤ 0.01. 

## Figures and Tables

**Table 1:** Infection protocols for *Cryptosporidium parvum* propagation in HCT-8 and COLO-680N cells in vitro.

Infection Protocol	Excystation Medium	Excystation Time	Infection Medium	Infection Time *	Agitation	Reference
**I**	100 μL of 0.01% trypsin and 400 μL of 0.5% sodium hypochlorite	1 h	RPMI 1640	24 h	intermittent vortexing (every ~10 min)	Miller et al., 2018
**II**	RPMI 1640 NaTC (0.4%) with pre-bleached oocysts	3 h	RPMI 1640 + NaTC (0.4%)	3 h	-	Shahiduzzaman et al., 2009
**III**	acidified (pH of 2.0) and non-acidified 1x HBSS	20 min	RPMI 1640	3 h	During the whole excystation time **	Varughese et al., 2014

The three protocols included washing steps at the end of infection time (thrice), using pre-warmed (37 °C) 500µL 1X  PBS. * Infection time stands for the period from sporozoite supplementation to host cells until the next medium change. (-) was not used. (NaTC) sodium taurocholate. ** Agitation was not used in the reference study; this step was added in the present study.

## Data Availability

Not applicable.
